# Liraglutide-Induced Pancreatitis: A Case Report and Literature Review

**DOI:** 10.7759/cureus.38263

**Published:** 2023-04-28

**Authors:** Hamna Javed, Gowthami Sai Kogilathota Jagirdhar, Rahul Kashyap, Pratikkumar H Vekaria

**Affiliations:** 1 Internal Medicine, Lahore Medical and Dental College, Lahore, PAK; 2 Research, Harvard Medical School, Boston, USA; 3 Internal Medicine, Saint Michael's Medical Center, Newark, USA; 4 Critical Care Medicine, Mayo Clinic, Rochester, USA; 5 Internal Medicine, Prisma Health University Medical Group, Greenville, USA

**Keywords:** glucagon-like peptide-1 receptor agonist (glp-1 ra), complications, diabetes mellitus type 2, pancreatitis, liraglutide

## Abstract

Liraglutide is an anti-diabetic medication used for the treatment of type 2 diabetes mellitus, obesity, and chronic weight management. It is a glucagon-like peptide-1 (GLP-1) agonist that helps reduce postprandial hyperglycemia for up to 24 h after administration. It stimulates endogenous insulin secretion according to glucose levels, and also delays gastric emptying and suppresses prandial glucagon secretion. Some of the common complications associated with liraglutide include hypoglycemia, headache, diarrhea, nausea, and vomiting. Uncommon adverse effects include pancreatitis, kidney failure, pancreatic cancer, and injection site reactions. In this article, we discussed a case of a 73-year-old male with a history of uncontrolled type 2 diabetes mellitus on long-term insulin and liraglutide who presented with abdominal pain, subjective fevers, dry heaves, tachycardia, and mildly reduced oxygen saturation. The patient was diagnosed with pancreatitis on the basis of laboratory and imaging findings. Liraglutide was discontinued, and the patient received supportive care with significant clinical improvement. The use of GLP-1 inhibitors has been increasing not only for diabetes mellitus management, but also for its promising effect on weight management. The literature review endorses our case report findings, and also discusses other complications of liraglutide. Therefore, we recommend to be cognizant of these side-effects upon starting liraglutide.

## Introduction

Liraglutide-induced pancreatitis has been reported through multiple case reports. Historically the treatment of diabetes mellitus has evolved with the discovery of insulin and modern methods of production making it widely available. The use of other anti-hyperglycemic agents has further revolutionized the management of not just diabetes but obesity as well. Per studies, along the lines of the cause of acute pancreatitis include gallstones, alcohol, or idiopathy [1]. However, many medications used for the treatment of diabetes have been studied as causative agents of pancreatitis including metformin [2] and empagliflozin [3]. Exenatide has been noted to cause a six-fold increase in the risk of acute pancreatitis in the post-marketing studies [4]. Exenatide and liraglutide are both glucagon-like receptor 1 (GLP-1) agonists, and have similar adverse reactions. Clinical trials by Novo Nordisk showed an incidence of 1.6 cases of acute pancreatitis per 1000 patient-years exposure to liraglutide compared to 0.7 cases per 1000 patient-years exposure for total active comparators [5]. The case we reported will support the relationship of liraglutide as the causative agent for acute pancreatitis.

## Case presentation

**Figure 1:**
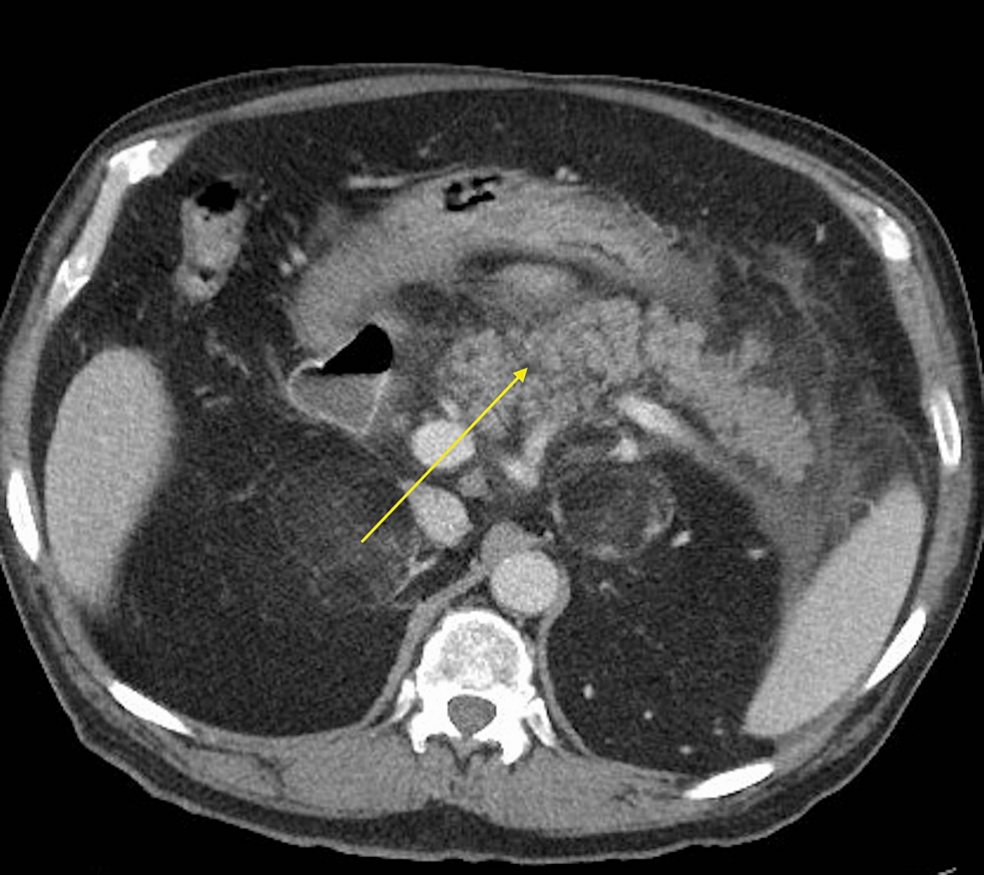
CT scan of abdomen and pelvis with contrast. Arrow shows diffuse edematous inflammation of pancreatic head, body, and tail

A 73-year-old male with a past medical history of uncontrolled type 2 diabetes mellitus on long-term insulin and liraglutide presented to the emergency department with the complaint of abdominal pain localized in the epigastric region associated with dry heaves and subjective fevers. The patient denied diarrhea, constipation, dysuria, recent travel, sick contacts, or consumption of fast food. He refused the use of alcohol, tobacco, or illicit drugs. The patient was being managed for uncontrolled diabetes mellitus with long-acting insulin, liraglutide, and metformin. The patient was on liraglutide for around 20 months before this admission. Physical examination was remarkable for diffuse abdominal tenderness that was most prominent in the epigastric region. Upon admission, vital signs were significant for a temperature of 99 degrees Fahrenheit, a pulse of 111 per min, blood pressure of 163/80 mmHg, and oxygen saturation of 90%-94% on room air. Laboratory work-up was notable for leukocytosis with a white blood cell count of 13,100/ µL, and an elevated serum lipase of 3389 U/L. An ultrasound of the right upper quadrant of the abdomen showed no evidence of cholecystitis or biliary dilation. Further workup with a CT scan of the abdomen and pelvis with contrast showed acute interstitial edematous pancreatitis (Figure 1).

Acute pancreatitis was considered the top differential because of the abdominal pain, elevated lipase levels (>3 times the upper limit of normal), and characteristic findings on CT scan of the abdomen and pelvis. He was placed on bowel rest and aggressive IV hydration. His liraglutide was discontinued and sliding scale insulin every 6 h was started. The patient showed improvement in abdominal pain and resolution of nausea. Improvement was also noted in the laboratory findings as mentioned in Table 1. A clear liquid diet was ordered for the patient which was advanced per the patient’s tolerance. The patient was advised to stop using liraglutide. The patient did not report any recurrence of acute pancreatitis on 10 months follow-up call.

**Table 1:** Trend in the patient's laboratory test results. WBC, white blood cells; Hgb, hemoglobin; AST, aspartate aminotransferase; ALT, alanine transaminase

Laboratory findings	Day 1	Day 2	Day 3	Day 5
WBC (x 10^9^/L)	13.1	22.0	18.3	9.6
Hgb (g/dL)	13.6	12.7	11.8	10.8
Platelets (x 10^9^/L)	389	295	261	311
Sodium (mEq/L)	138	134	138	138
Potassium (mEq/L)	4.8	4.1	4.0	3.6
Serum creatinine (mg/dL)	1.30	1.20	1.27	1.14
Total Bilirubin (mg/dL)	0.7	0.8	0.5	
AST (IU/L)	23	23	19	18
ALT (IU/L)	15	13	8	10
Alkaline phosphatase (IU/L)	45	47	50	63
Lipase (U/L)	3389	1147	392	
Calcium (mg/dL)	9.5	8.8	8.7	7.8
Triglyceride (mg/dL)	115			

## Discussion

Acute pancreatitis is characterized as the inflammation of the pancreas in a rapid time frame. It can have many underlying etiologies, however, the occurrence due to side effects of drugs is still being studied. An analysis of 713 cases implicated 213 unique drugs as a causative agent [6]. Liraglutide is commonly used in diabetic patients particularly to aid in weight loss for overweight/obese patients. Literature on the observation of post-marketing effects and pre-marketing reports of liraglutide-related pancreatitis, as well as the incidence of symptoms after the initiation of liraglutide, is highly suggestive of a relationship [5, 7]. The GLP-1 agonist impact on the biliary system has been well studied with conclusions that the mechanism of causing pancreatitis is its ability to reduce biliary and gallbladder motility which has resulted in cholelithiasis in many patients [8]. However, the occurrence of pancreatitis has been noted with the absence of cholelithiasis. The results are thought to be due to liraglutide's ability to expand the pancreatic glands [9]. Eight cases of incretin-induced pancreatitis were seen during clinical development, and 36 cases were in the post-marketing phase [10]. In a systematic review and meta-analysis of six observational studies, conducted on incretin-based therapies in acute pancreatitis, no significant difference was found in the overall risk of pancreatitis between incretin users and non-users. Presumably, the patients who have already been on the medications with an established high risk of developing pancreatitis with or without alcohol use, and are now on liraglutide are more susceptible to develop pancreatitis compared to the general population. The conflicting results in current evidence necessitate further large-scale observational studies on the effect of GLP-1 receptor agonists in causing acute pancreatitis.

However, with incidences being reported it is safe to say that an association is present between the use of liraglutide and acute pancreatitis. The patient that we discussed in the case report above had no other discernable cause for acute pancreatitis. The association was made after ruling out the other causes. No recurrence was noted in follow-up after discontinuation of liraglutide.

Literature review

Using the search criteria of (Liraglutide) AND (pancreatitis) AND (Case report OR Case series) on PubMed with a case report filter, we found 26 case reports. After the exclusion of the articles that were not case reports and were unrelated cases, we ended up with 12 relevant cases for this literature review (Table 2). The mean age of the patients was noted to be 51.67 years with a range of 25-75 years. It was notable that five were males and seven were females.

**Table 2:** Previously published case reports on GLP-1 agonist induced pancreatitis. RUQ, right upper quadrant; LUQ, left upper quadrant; QD, once a day; PMH, past medical history; ACE-i, angiotensin converting enzyme inhibitor; US, ultrasound; ESRD, end stage renal disease; NPO, nil per os; IV, intravenous; TGL, triglyceride

Author	Age	Gender	Complaints	Dose of Liraglutide	Duration (months)	Treatment	Comorbidities/PMH	Medications	Lipase	TGL	CT findings
Lee et al. [7]	60	F	Mid-epigastric abdominal pain	1.8 mg QD	0.69	Discontinued liraglutide, conservative therapy	Type 2 diabetes mellitus	Exenatide, Liraglutide	478	None	None
Dolan et al. [9]	31	M	Sharp mid-epigastric pain radiating to the back and left upper abdomen	3 mg QD	10	Discontinued liraglutide, IV fluids (ringer lactate)	Type 2 diabetes mellitus	Levothyroxine, Liraglutide	156	279	Mild interstitial pancreatitis on CT scan
Korkmaz et al. [13]	75	F	Abdominal pain	None noted	6	NPO, IV fluids, ceftriaxone, discontinued liraglutide	Cholelithiasis	Liraglutide	None	None	Acute cholecystitis and cholelithiasis
Famularo et al. [14]	67	M	Nausea, vomiting and epigastric pain	1.2 mg QD	5	Discontinued liraglutide, bowel rest, IV fluids	Type 2 diabetes mellitus	Metformin, Gliclazide, Liraglutide	653	None	MRI showed moderately enlarged and edematous pancreas and sludge in the gallbladder
Nafisah et al. [15]	28	F	Epigastric pain radiating to back	18 mg	None	Discontinued liraglutide, IV dextrose	Type 2 diabetes mellitus, obesity	Metformin, Multivitamin, Liraglutide	None	None	None
Madsen and Christiansen [16]	43	M	Nausea and vomiting	54 mg once	None	IV fluids, bowel rest	Type 2 diabetes mellitus	Metformin, ACE-i, Liraglutide	None	None	None
Nakata et al. [17]	75	F	Nausea	0.6 mg	6	No specific treatment plan was reported	Type 2 diabetes mellitus, ESRD	Vildagliptin, Cilostazol, Calcium carbonate, Liraglutide	306	111	None
	68	M	Nausea	None noted	None	No specific treatment plan was reported	Type 2 diabetes mellitus, ESRD	Liraglutide	None	None	None
AlSaadoun et al. [18]	25		Epigastric abdominal pain radiating to the back	3 mg QD	2	Bowel rest, analgesia, IV fluids, antibiotics	Type 2 diabetes mellitus, cholelitheasis	Liraglutide	> 900	None	Unremarkable US
Knezevich et al. [19]	53	M	RUQ and LUQ abdominal pain	1.2 mg QD	2	Discontinued liraglutide	Hyperlipidemia, hypertension, peripheral neuropathy, erectile dysfunction, and obesity	Aspirin, Metformin, Simvastatin, Tadalafil, Glimepiride, Liraglutide	>1500	None	Acute pancreatitis on radiographs
Jeyaraj et al. [20]	51	F	Abdominal pain, nausea and vomiting	None noted	1.8	No specific treatment plan was reported	Type 2 diabetes mellitus	Liraglutide	None	None	CT showed pancreatitis
Quesada-Vasquez [21]	44	F	Epigastric pain radiating to back	1.2 mg QD	6	Conservative management	Type 2 diabetes mellitus	Liraglutide	None	None	None

The most common presenting symptom was epigastric abdominal pain followed by nausea and vomiting. The mean dose of liraglutide was 1.61 mg daily with a range of 0.6-3 mg of daily dose. The duration of liraglutide before the onset of the pancreatitis ranged between 0.69 and 10 months from the day of introduction. The patients were all noted to have type 2 diabetes mellitus or obesity for which the liraglutide was prescribed. Most of the cases had a noted finding of pancreatitis on a CT scan. If mentioned, lipase levels were elevated at least three times the upper limit of normal. There were no recent changes in the dosage of liraglutide in any of the mentioned case reports. The common management for these patients was discontinuing the liraglutide and supportive care with IV fluids, and pain management. Most of the patients recovered without complications from this management. Two of the 12 cases had cholelithiasis which may be a contributing and/or confounding factor to pancreatitis. A total of four patients were concurrently taking metformin along with liraglutide.

No significant short or long-term complications were noted except hemorrhagic pancreatitis. There were also noted to have recurrent episodes of pancreatitis, acute respiratory distress syndrome, and acute kidney injury [11]. It was noted that gastrointestinal symptoms of pancreatitis were the most frequently reported adverse reactions followed by renal, hepatic, and immunological complications [12]. Patients were able to make a complete recovery in all the cases and were advised to avoid the use of liraglutide as it was assumed to be the most likely etiology for pancreatitis. Acute pancreatitis due to liraglutide overdose was also noted in two cases that were resolved with conservative management and discontinuation of the offending medication. Literatures did not show any significant dose-effect relationship. The cases arose due to misunderstanding in frequency and dose to be self-administered. Table 2 describes further details on the 12 case reports that we included in this literature review.

The limitations of our case report and literature review include the limited availability of data which precludes us from conducting a thorough retrospective case-control study. Also, we did not have data on all the pre-existing conditions and laboratory reports that could have contributed to the induction of acute pancreatitis. We have only included the cases found on the PubMed database written in the English language which may lead to missing data on the cases written in a different language. 

## Conclusions

Liraglutide is an anti-diabetic medication that can cause acute pancreatitis as a rare complication. Pancreatitis from liraglutide can occur as early as three weeks after starting the medication. Other risk factors for pancreatitis such as metformin and cholelithiasis can further predispose to pancreatitis development while on liraglutide. Awareness of this complication aids in early diagnosis and stoppage of the offending medication to prevent complications and recurrence of pancreatitis.
